# Ultrasound for the Early Detection and Diagnosis of Necrotizing Enterocolitis: A Scoping Review of Emerging Evidence

**DOI:** 10.3390/diagnostics15151852

**Published:** 2025-07-23

**Authors:** Indrani Bhattacharjee, Michael Todd Dolinger, Rachana Singh, Yogen Singh

**Affiliations:** 1 Department of Pediatrics, Tufts University School of Medicine, Boston, MA 02111, USA; indrani.bhattacharjee@tuftsmedicine.org (I.B.); rachana.singh1@tuftsmedicine.org (R.S.); 2 Division of Pediatric Gastroenterology, NYU Grossman School of Medicine, New York, NY 10016, USA; michael.dolinger@nyulangone.org; 3 Department of Pediatrics, Division of Neonatology, UC Davis Children’s Hospital, University of California, 2516 Stockton Blvd, TICON-II, Sacramento, CA 95817, USA

**Keywords:** necrotizing enterocolitis, neonatal ultrasound, Doppler imaging, POCUS, neonatal imaging, diagnostic accuracy, artificial intelligence, bowel perfusion, early detection

## Abstract

**Background:** Necrotizing enterocolitis (NEC) is a severe gastrointestinal disease and a major cause of morbidity and mortality among preterm infants. Traditional diagnostic methods such as abdominal radiography have limited sensitivity in early disease stages, prompting interest in bowel ultrasound (BUS) as a complementary imaging modality. **Objective:** This scoping review aims to synthesize existing literature on the role of ultra sound in the early detection, diagnosis, and management of NEC, with emphasis on its diagnostic performance, integration into clinical care, and technological innovations. **Methods:** Following PRISMA-ScR guidelines, a systematic search was conducted across PubMed, Embase, Cochrane Library, and Google Scholar for studies published between January 2000 and December 2025. Inclusion criteria encompassed original research, reviews, and clinical studies evaluating the use of bowel, intestinal, or Doppler ultrasound in neonates with suspected or confirmed NEC. Data were extracted, categorized by study design, population characteristics, ultrasound features, and diagnostic outcomes, and qualitatively synthesized. **Results:** A total of 101 studies were included. BUS demonstrated superior sensitivity over radiography in detecting early features of NEC, including bowel wall thickening, portal venous gas, and altered peristalsis. Doppler ultrasound, both antenatal and postnatal, was effective in identifying perfusion deficits predictive of NEC onset. Neonatologist-performed ultrasound (NEOBUS) showed high interobserver agreement when standardized protocols were used. Emerging tools such as ultra-high-frequency ultrasound (UHFUS) and artificial intelligence (AI)-enhanced analysis hold potential to improve diagnostic precision. Point-of-care ultrasound (POCUS) appears feasible in resource-limited settings, though implementation barriers remain. **Conclusions:** Bowel ultrasound is a valuable adjunct to conventional imaging in NEC diagnosis. Standardized protocols, validation of advanced technologies, and out come-based studies are essential to guide its broader clinical adoption.

## 1. Introduction

Necrotizing enterocolitis (NEC) is the most serious gastrointestinal complication with significantly high morbidity and mortality in preterm infants [1,2]. This acute inflammatory condition affects the intestines of preterm infants, with an incidence estimated between 4% and 10% in very-low-birth-weight (VLBW) infants born before 32 weeks of gestation [3,4]. Despite notable progress in neonatal intensive care practices, NEC continues to carry a significant mortality risk, with reported death rates ranging from 30% to 50%, especially with surgical NEC [5,6].

NEC is a complex disease with multifactorial pathogenesis, characterized by a rapid and often unpredictable onset. The diseases’ inherent pathophysiologic complexity and etiological heterogeneity make creating models for primary research problematic. A wide range of contributing factors has been identified, including prematurity, genetic susceptibility, exposure to chorioamnionitis, perinatal hypoxia, milk feeding types, viral infections, disruptions in gut microbiota, and severe anemia requiring transfusion of red blood cells [6,7,8]. This makes the diagnosis of NEC difficult, as the clinical presentation seen with NEC may also be seen with other benign conditions, which then erroneously are placed under the broader umbrella of NEC [5,9,10,11].

Currently, the mainstay of NEC diagnosis relies on symptomatology, clinical assessment abdominal radiography, and post-surgical histopathology. The widespread availability of portable X-ray machines, their cost-effectiveness, and the familiarity of clinicians with interpreting radiographic findings have reinforced the continued use of abdominal radiographs in the workup of suspected NEC. However, even under optimal conditions, radiographic techniques are estimated to detect only about 55–60% of NEC cases [11]. Radiographic uncertainty in diagnosing NEC remains a critical and widely acknowledged limitation in neonatal care. Despite being the conventional imaging modality, abdominal X-rays (AXRs) are often fraught with interpretive ambiguity, particularly in early or equivocal cases of NEC. This diagnostic uncertainty can significantly delay interventions or, conversely, lead to overtreatment. A landmark study by Cuna et al. highlighted that radiologists reported significantly higher levels of uncertainty when interpreting AXRs compared to bowel ultrasound (BUS), underscoring the subjective nature and variability in plain radiograph interpretation [12].

Mounting evidence demonstrates that AXRs often fail to detect early or subtle signs of disease, such as focal bowel wall thickening, reduced perfusion, or localized inflammatory changes—features critical for timely diagnosis and intervention [13,14]. Moreover, the interpretive reliability of AXRs is compromised by projectional overlap, ambiguous gas patterns, and high inter-reader variability, leading to frequent discrepancies in clinical decision-making [15,16]. As highlighted by Kim et al. and others, the static and nonspecific nature of radiographic findings makes AXR poorly suited for capturing the evolving and dynamic pathology of NEC [17]. Taken together, these limitations underscore that continued reliance on AXR alone not only delays diagnosis but also perpetuates clinical ambiguity—particularly in the earliest and most vulnerable stages of the disease. There exists an urgent need to have more accurate diagnostic techniques, including radiological methods, to make an early diagnosis of NEC to improve infant outcomes.

More recently, ultrasound imaging has gained traction as a promising tool in the evaluation of NEC. The increasing availability of compact, handheld ultrasound devices has improved the feasibility of bedside gastrointestinal assessment. These devices are easily transportable, capable of generating high-resolution images, and allow for frequent monitoring without subjecting infants to ionizing radiation.

## 2. Rationale for This Scoping Review

Despite the increasing use of ultrasound in the diagnosis of NEC, standardized protocols and consensus guidelines are lacking, leading to variability in clinical practice. This scoping review aims to summarize the existing literature on the role of ultrasound in NEC diagnosis, compare ultrasound with traditional imaging modalities such as X-ray and computed tomography (CT) and assess the strengths, limitations, and future directions for integrating ultrasound into NEC management.

## 3. Methodology

### 3.1. Study Design

**Figure 1:**
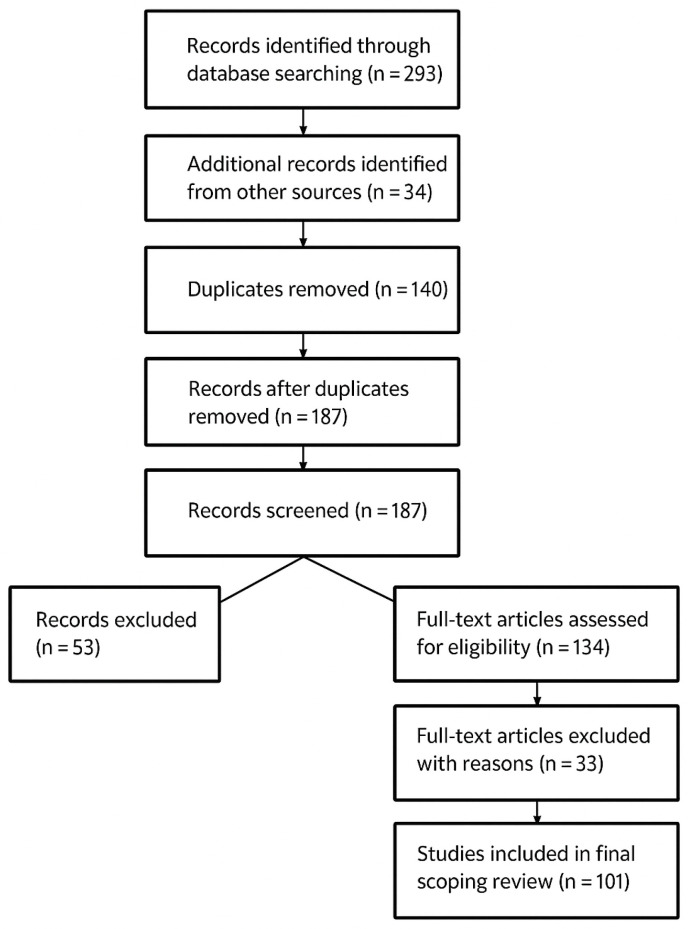
PRISMA flow diagram of study selection process.

This scoping review was conducted using the Preferred Reporting Items for Systematic Reviews and Meta-Analyses (PRISMA-ScR) guidelines (Figure 1). A scoping review methodology was chosen due to its ability to map the existing literature, identify research gaps, and summarize diverse study designs related to the role of ultrasound in the diagnosis and management of NEC. A systematic literature search was conducted to evaluate the use of bowel, intestinal, and abdominal ultrasound in the diagnosis of NEC in neonates.

### 3.2. Eligibility Criteria

Studies were selected based on the following inclusion and exclusion criteria:

Inclusion Criteria: Randomized controlled trials (RCTs), observational studies, case series and meta-analyses, and “studies including preterm and term newborns with suspected or confirmed NEC” published in peer-reviewed journals between January 2000 and December 2025, with available or translated abstracts in English, examining the role of ultrasound (abdominal, intestinal, intestinal or Doppler) in the diagnosis of NEC.

### 3.3. Search Strategy

A comprehensive search was conducted across the following databases:

- PubMed (MEDLINE)
- Embase
- Cochrane Library
- Google Scholar

The search strategy included MeSH terms and keywords related to NEC and ultrasound:

(“Enterocolitis, Necrotizing” OR necrotizing enterocolitis) AND (“Infant, Newborn”] OR neonate* OR “Infant, Premature”) AND (“Ultrasonography” OR “Ultrasonography, Doppler” OR ultrasound OR “Point-of-Care Systems” AND (“Intestines” OR bowel OR intestinal) AND (“Diagnosis “OR “Monitoring, Physiologic” OR assessment OR prognosis*)

Search filters were applied to include studies published between January 2000 and December 2025.

### 3.4. Study Selection Process

- Step 1: Title/Abstract Screening—Two independent reviewers screened all retrieved articles.
- Step 2: Full-Text Review—Selected studies underwent detailed evaluation based on inclusion criteria.
- Step 3: Data Extraction and Charting—Relevant information was compiled into a standardized data extraction sheet.
- The full search strategy and PRISMA-ScR checklist are available in the Supplementary Materials.

### 3.5. Data Extraction and Categorization

The extracted data were organized into key study characteristics including the following:

1. Study Design—RCT, cohort, case-control, systematic review.
2. Sample Population—Gestational age, birth weight, NEC severity.
3. Ultrasound Indications—Screening, diagnosis, disease progression.
4. Diagnostic Findings—Sensitivity, specificity, ultrasound markers.
5. Comparison with X-ray and Other Imaging—Accuracy and clinical impact.

### 3.6. Data Synthesis

- Studies were categorized into summary tables and figures based on study design, population, indications, and outcomes.
- Findings were synthesized qualitatively, emphasizing ultrasound’s clinical role, strengths, and limitations.

The methodological quality of the included studies was rigorously assessed using the Medical Education Research Study Quality Instrument (MERSQI). This evaluation focused on the study design, methodology, sample size, reliability and validity of assessment tools, statistical analysis methods, and the relevance of outcomes to educational objectives. This quality assessment helped identify the strength of evidence and potential biases in the included studies.

## 4. Results

A comprehensive literature review was conducted to identify studies relevant to the research question. The initial search yielded a total of 293 records through electronic database searches. In addition, 34 records were identified through other sources such as manual searches of reference lists and grey literature, resulting in a total of 327 records.

After removing 140 duplicate records, 187 unique records remained and were subjected to title and abstract screening. Of these, 53 records were excluded based on irrelevance to the study objectives, leaving 134 articles for full-text review. Upon detailed evaluation of the full texts, 33 articles were excluded for reasons such as inappropriate study design, population mismatch, or lack of relevant outcome measures. Ultimately, 101 studies were included in the final scoping review. The process of study selection is illustrated in the PRISMA flow diagram (Figure 1)

### 4.1. Distribution by Study Design

A summary of designs of the included studies and their clinical relevance:

The majority of studies were cohort designs, likely reflecting the predominance of observational approaches in NEC research. The limited number of randomized controlled trials (RCTs) underscores the inherent difficulties of conducting trials in critically ill neonatal populations as illustrated in Table 1.

### 4.2. Study Population (Gestational Age and Birth Weight)

The reviewed studies demonstrated that ultrasound findings in NEC cases varied significantly based on gestational age (Table 2). Among extremely preterm infants (<28 weeks, 45 studies), NEC was often severe and rapidly progressive. Doppler ultrasound commonly revealed absent or reversed diastolic flow in the superior mesenteric vessels, indicative of intestinal ischemia, necessitating serial ultrasound monitoring to assess disease progression. In the very preterm group (28–32 weeks, 35 studies), NEC presentation was more variable, with some cases resolving spontaneously while others progressed to severe disease. Doppler assessments suggested that early perfusion abnormalities could serve as predictors for later NEC progression, highlighting the potential role of ultrasound in early risk stratification. In contrast, late preterm and term infants (>32 weeks, 20 studies) experienced milder NEC, often associated with underlying comorbidities such as congenital heart disease. While these cases exhibited fewer ultrasound findings of bowel wall thickening, they demonstrated a higher prevalence of portal venous gas, differentiating ultrasound findings from those seen in NEC in preterm neonates. These findings underscore the importance of gestational age-specific ultrasound criteria to enhance the early detection, risk assessment, and management of NEC (Table 2).

### 4.3. Indications for Ultrasound Use

In extremely preterm infants (<28 weeks), ultrasound findings tend to reflect the severe immaturity of the intestinal wall, vasculature, and immune interface, with bowel wall abnormalities and perfusion deficits appearing early and often preceding clinical deterioration. Duci et al. [18]. demonstrated that fetal Doppler evaluation, particularly absent or reversed end-diastolic flow in the superior mesenteric artery (SMA), served as a significant early indicator of intestinal ischemia, a precursor to NEC. This was supported by Guang et al., who found that in infants born before 28 weeks, diminished or reversed diastolic flow in the SMA detected via early Doppler ultrasound was strongly associated with subsequent NEC diagnosis, with an increased predictive value compared to standard radiographs. In these infants, classic ultrasound findings such as bowel wall thickening, absent peristalsis, and portal venous gas appeared earlier in the disease process and were more pronounced than in older gestational age groups at birth, aligning with the underlying pathophysiological vulnerability of the immature gut. A recent systematic review of eight studies involving 494 neonates, including 126 with NEC, found that Doppler indices measured on the first postnatal day were significantly different in infants who later developed NEC compared to controls. In contrast, these markers were less predictive once NEC had already developed. Notably, 75% of NEC cases in one subgroup occurred within the first 24 h of life—before feeding began—underscoring Doppler ultrasound’s critical role in facilitating early, preclinical intervention [19]. The predictive power of ultrasound in this population is heightened by the rapid progression of NEC and the window for early intervention is narrow; thus, high-resolution and Doppler-based imaging is crucial in guiding management [20,21].

Nakayuenyongsuk et al. [22], amongst others, observed that in very preterm infants (28–32 weeks), ultrasound findings such as bowel wall thickening and persistent bowel loop dilatation were common, but less specific. Notably, when these imaging markers were combined with fecal calprotectin measurements, diagnostic accuracy improved significantly. The addition of biomarker monitoring helped bridge the sensitivity gap seen in ultrasound alone for this age group, suggesting that multimodal approaches are better suited to capture the heterogeneous presentation of NEC in maturing intestines. Unlike in extremely preterm neonates, Doppler flow in this group showed variable changes and was not consistently associated with adverse outcomes, indicating that vascular compromise be less central to NEC pathogenesis at this developmental stage [18,23,24].

In late preterm and term infants (>32 weeks), the ultrasound features diverge further, with vascular Doppler findings becoming even less reliable as early markers of NEC. Instead, May et al. emphasized the importance of recognizing portal venous gas, complex free peritoneal fluid, and focal bowel wall echogenicity as critical markers in more mature infants. These findings, while also present in extremely preterm infants, were more often observed later in the disease course in older gestational ages, when clinical signs had already emerged. Importantly, the authors stressed that in this group, NEC often presents atypically, with less prominent bowel wall thickening and preserved Doppler flow until advanced stages, making ultrasound a supportive, rather than primary, diagnostic modality. This shift highlights the relative resilience of the term bowel to ischemia and underscores the need for careful correlation with clinical presentation [16].

Singh et al. provided a broader translational view, reinforcing that NEC pathogenesis shifts with gestational age, which in turn impacts the sonographic profile. In extremely preterm infants, the disease is more likely to be driven by hypoxic perfusion failure and immaturity of the epithelial barrier, which translates into earlier and more prominent ultrasound findings. Conversely, in older preterm and term infants, NEC may follow a more localized inflammatory pattern, leading to subtler imaging changes and often requiring adjunct diagnostic support, such as laboratory markers or cross-sectional imaging [25].

### 4.4. Key Sonographic Markers in the Diagnosis of NEC

Ultrasound has demonstrated superior sensitivity and clinical utility over abdominal radiographs in the evaluation of NEC, particularly for early detection and risk stratification. [26]. Bowel wall thickening (>2.5–3 mm), a key early sonographic indicator of intestinal inflammation, is frequently visible before radiographic changes occur and is summarized in Table 3 for its diagnostic relevance compared to X-ray. Kim et al. found that ultrasound detected bowel wall thickening in early-stage NEC with greater accuracy than X-ray, which often lacks specificity at this stage. This early indicator is particularly valuable in extremely preterm infants, where intervention timing is critical [17].

Pneumatosis intestinalis, a hallmark of NEC, is traditionally detected on radiographs, yet studies have shown ultrasound to be more sensitive, especially in early disease. Muchantef et al. and May et al. emphasized that ultrasound often reveals intramural gas before it becomes radiographically evident. These findings align with the work of Bohnhorst, who concluded that ultrasound offers better real-time visualization of gas within the bowel wall, particularly in non-classic presentations of NEC [16,24,27]

Importantly, decreased or absent peristalsis—a marker of NEC progression—is uniquely detectable by ultrasound. This dynamic assessment allows clinicians to identify functional bowel compromise, which cannot be assessed by static radiographs. Hwang et al. and Lazow et al. linked absent peristalsis with higher rates of surgical NEC, showing its value in the early escalation of care. The integration of peristalsis evaluation into NEC scoring systems has been proposed by several authors, including Zarei et al., as a critical component in predicting disease severity [21,28,29].

Portal venous gas (PVG), often underdiagnosed on X-ray, but is reliably detected on ultrasound as hyperechoic foci within the liver or portal branches. Guang et al. and Singh et al. linked PVG to poor prognosis and higher surgical risk. The earlier detection of PVG with ultrasound allows for timelier decisions around conservative versus operative management, particularly in borderline cases [20,25].

The presence of free intraperitoneal fluid—especially complex or echogenic fluid—is a predictor of perforation or severe intestinal compromise. While X-ray may detect free fluid in later stages, ultrasound excels at detecting early fluid accumulation. Palleri et al. [30] demonstrated that complex fluid on ultrasound strongly correlates with the need for surgery. This has been reaffirmed in clinical scoring models by Lazow et al. and Zarei et al., who identified fluid characteristics as key predictors of adverse outcomes [21,23,29]

Finally, Doppler ultrasound assessment of bowel wall perfusion offers a dimension entirely absent from radiographic imaging. Several studies confirm that absent or reversed flow in the superior mesenteric artery is predictive of NEC severity and progression, especially in extremely preterm neonates. This perfusion insight not only aids diagnosis but also assists in triaging for surgical consultation [19,22,27,31].

A truly novel modality is ultra-high-frequency ultrasound (UHFUS), operating between 30 and 100 MHz. This technology offers exceptional spatial resolution, enabling earlier detection of bowel wall abnormalities and ischemic changes than conventional ultrasound or X-rays. In a feasibility study, Jacobsen et al. demonstrated that UHFUS could visualize intestinal microvasculature and distinguish early NEC from other bowel conditions—opening new possibilities for real-time, microstructural gut assessment [32].

### 4.5. Evolution of Ultrasound Use in NEC

**Figure 2:**
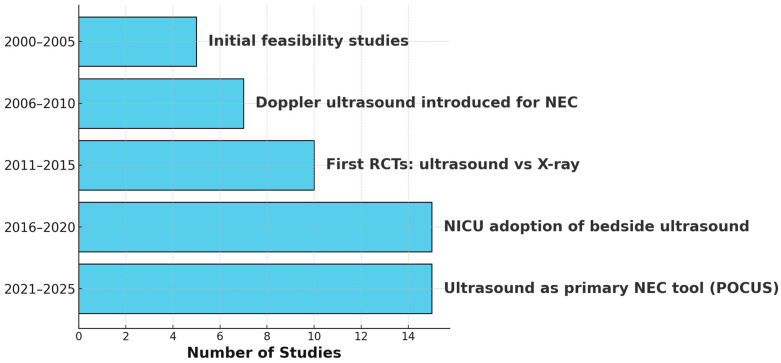
Timeline illustrating the evolution of ultrasound research in necrotizing enterocolitis (NEC) from 2000 to 2025.

Ultrasound research in the context of NEC has evolved significantly over the past two decades. Early studies between 2000 and 2005 focused on establishing the feasibility of using ultrasound in neonatal intestinal imaging. This was followed by the introduction of Doppler ultrasound for assessing bowel perfusion between 2006 and 2010. The period from 2011 to 2015 saw the emergence of randomized clinical trials comparing ultrasound to traditional radiography, marking a pivotal moment in the validation of ultrasound as a diagnostic tool. From 2016 to 2020, the adoption of bedside ultrasound in neonatal intensive care units (NICUs) accelerated, reflecting growing clinical acceptance. Most recently, between 2021 and 2025, ultrasound—particularly point-of-care ultrasound (POCUS)—has gained recognition as a primary modality for the diagnosis of NEC. This progression in both the volume of research and the depth of clinical application is depicted in Figure 2, which illustrates the timeline of ultrasound research in NEC from 2000 to 2025. These key developments not only represent a shift in research focus but also indicate increasing reliance on ultrasound for early, dynamic, and bedside evaluation of neonatal gut health. This progression in both the volume of research and the depth of clinical application is depicted in the figure below.

To further illustrate the scope and impact of the current literature, Table 4 summarizes selected high-impact studies that exemplify the diagnostic applications of ultrasound in NEC. These studies represent a range of methodologies—from traditional Doppler assessments to AI-integrated ultrasound and ultra-high-frequency imaging—highlighting the evolving landscape of sonographic evaluation in neonatal intestinal pathology.

## 5. Discussion

The use of ultrasound in the diagnosis and management of NEC has gained increasing attention in neonatal care, with numerous studies evaluating its advantages and limitations compared to conventional imaging modalities. Historically, abdominal radiographs have been the primary imaging modality for NEC diagnosis. However, their limited sensitivity in early-stage NEC, particularly in cases where pneumatosis intestinalis is absent, has prompted the exploration of alternative diagnostic tools [17,28]. While radiography remains a standard in neonatal intensive care units (NICUs), studies suggest that ultrasound provides higher diagnostic accuracy by enabling real-time visualization of bowel wall integrity, vascular perfusion, and early-stage inflammation [14,35].

### 5.1. Operator Dependency and Training Challenges: Neonatologist-Performed Bowel Ultrasound: NEOBUS

Bowel ultrasound (BUS) performed by neonatologists is an emerging tool for NEC diagnosis. However, until recently there was little data on consistency amongst neonatologists in interpreting ultrasound findings. In 2018, a European multi specialist survey gathered responses from 202 neonatologists, pediatric surgeons, and radiologists to assess current practices and perceptions regarding imaging in NEC. It revealed strong agreement with over 90% of respondents agreeing that abdominal X-rays are essential for confirming NEC, monitoring progression, and guiding surgical decisions. Only 58% of clinicians reported using ultrasound for NEC diagnosis [11]. This implied that, unlike radiologists, neonatologists had not been formally studied for consistency in ultrasound interpretations.

A growing body of evidence suggests that neonatologists can achieve high interobserver agreement in BUS interpretation when using standardized approaches. In a 2024 prospective study by Kimble et al. involving 10 preterm infants, a neonatologist and a pediatric radiologist independently assessed bowel content using a novel ultrasound scoring system [36]. The resulting kappa statistic of 0.91 indicated near-perfect agreement, reinforcing the reproducibility of such protocols. Similarly, Priyadarshi et al. and others highlighted strong interobserver concordance (~0.91) among neonatologists using standardized image scoring in their NICU, emphasizing that structured training enables reliable ultrasound assessments [37,38]. However, broader surveys and expert opinions caution that confidence and consistency in interpreting certain findings—such as pneumatosis—still vary across centers. These insights underline the critical need for uniform training and protocols to minimize variability and maximize the diagnostic value of ultrasound in NEC [12,34,39].

### 5.2. How Early Is Early: Doppler Ultrasound and Its Role in Early NEC Detection

Recent studies suggest that Doppler ultrasound, both antenatally and postnatally, may be a valuable tool in predicting and potentially preventing NEC in preterm infants. Duci et al. and others evaluated fetal Doppler indices—specifically in the superior mesenteric artery (SMA), umbilical artery (UA), and middle cerebral artery (MCA)—in growth-restricted fetuses during the third trimester. They found that absent or reversed end-diastolic flow (AREDF) in the SMA was strongly associated with later intestinal complications, including NEC, likely due to impaired gut perfusion. The altered splanchnic–cerebral Doppler ratio confirmed fetal blood flow redistribution, a “brain-sparing” effect in response to hypoxia. Guang et al. extended this concept postnatally, showing that abnormal SMA Doppler patterns (elevated resistance and pulsatility indices) detected within the first 72 h of life could precede clinical NEC. Their findings suggest early SMA Doppler screening may enable individualized risk stratification and proactive care. Supporting this, Moschino et al. linked abnormal mesenteric Doppler findings to NEC in neonates with congenital heart disease, while Ince et al. [40] emphasized the role of impaired postprandial SMA blood flow as a predictor of NEC. Similarly, Zhao et al. proposed a predictive nomogram incorporating Doppler findings for neonates with fetal growth restriction, potentially extendable to NEC risk models [41]. Collectively, these studies highlight the potential of Doppler ultrasound to guide early intervention strategies and feeding protocols to mitigate NEC risk [18,20,39,42].

### 5.3. POCUS Verses Standard US

POCUS has gained momentum as an effective bedside diagnostic tool in NICUs. The American Academy of Pediatrics (AAP) and European Society of Pediatric and Neonatal Intensive Care (ESPNIC) have published statements in favor of increased use of POCUS by neonatologists for multiple indications [43,44]. Unlike standard ultrasound, which requires radiology department involvement, POCUS enables real-time assessment by neonatologists, reducing delays in diagnosis and clinical decision-making. Specifically with respect to NEC, literature is emerging to support its use in evaluating neonatal bowel, such as a comparative study by Patel et al. demonstrating POCUS sensitivity for NEC detection of 88%, compared to 75% for standard ultrasound and 65% for radiographs [4,45].

### 5.4. Ultra-High-Frequency Ultrasound (UHFUS)

Jacobsen et al. and others demonstrated the novel use of UHFUS for visualization of bowel microvascular structures, enabling earlier identification of ischemic injury. The study found that UHFUS was able to detect NEC-related bowel changes before they were visible on standard ultrasound or X-ray, allowing for earlier medical interventions that could prevent disease progression. This study highlights UHFUS as a valuable addition to NEC diagnosis and screening, particularly in neonates with ambiguous clinical symptoms [32,46].

### 5.5. Integration of AI in NEC Diagnosis

The use of artificial intelligence (AI) has been evolving in ultrasound imaging to improve NEC detection and diagnostic precision. Yao et al. [33] reported that AI-assisted ultrasound interpretation increased NEC diagnostic accuracy by 15% compared to manual ultrasound interpretation, highlighting its potential to reduce human error. Wu et al. and others developed a deep learning model integrating ultrasound imaging and Doppler flow data, which outperformed traditional diagnostic methods in identifying early-stage NEC [34,47]. AI also enhances real-time image processing, allowing clinicians to detect subtle bowel perfusion abnormalities before visible damage occurs. Xie et al. [35] demonstrated that RFImageNet can use AI to segment ultrasound images with high accuracy, aiding in early NEC detection. These advancements underscore the transformative role of AI in enhancing the accuracy, efficiency, and timeliness of NEC diagnosis through ultrasound imaging in the coming years.

### 5.6. Long-Term Prognostic Value of Ultrasound in NEC

Recent studies highlight the importance of early and accurate diagnosis of NEC and its impact on neurodevelopmental outcomes in survivors. Research comparing ultrasound and X-ray-based NEC diagnosis reveals that infants diagnosed via ultrasound have significantly better cognitive and motor function than those diagnosed with X-ray. Mathews et al. [48] found that ultrasound detected NEC earlier and with greater sensitivity, allowing for timely intervention, reducing the severity of intestinal damage, and improving long-term neurological outcomes. In contrast, Soni et al. reported that 25% of NEC survivors diagnosed via X-ray suffered severe cognitive and motor impairments, highlighting the limitations of X-ray in early NEC detection [49]. Similarly, Garg et al. [5] demonstrated that severe NEC (Stage III) correlated with lower IQ scores, motor delays, and structural brain abnormalities, particularly in infants who underwent surgical intervention. Brain imaging showed higher rates of periventricular leukomalacia (PVL) in surgically treated NEC cases, further supporting the need for earlier detection and medical management when possible. Magnusson et al. followed NEC survivors to age five, revealing poorer neurodevelopmental outcomes in those diagnosed via X-ray compared to ultrasound-based diagnoses. The study reinforced that infants who had early-stage NEC cases detected by ultrasound had better motor skills and higher cognitive scores than infants with late-stage diagnoses. These findings emphasize the need to integrate ultrasound as a standard diagnostic tool for NEC, as it enables earlier intervention, reduces the risk of brain injury, and improves long-term developmental outcomes. Furthermore, AI-assisted ultrasound technology holds promise for enhancing NEC detection accuracy, potentially reducing the reliance on X-ray, which remains less sensitive for early NEC diagnosis. Overall, these studies advocate for a shift towards ultrasound-driven NEC diagnostics to optimize neurodevelopmental outcomes and improve quality of life in NEC survivors [5,43].

Beyond long-term outcomes, early diagnostic insights from ultrasound may also influence immediate clinical management. Early identification of NEC through ultrasound may allow clinicians to intervene before the onset of advanced disease. When sonographic findings such as bowel wall thickening, portal venous gas, or perfusion deficits are detected early, clinicians can escalate monitoring, initiate bowel rest, administer empirical antibiotics, and delay or modify enteral feeding. These preemptive steps may reduce the risk of progression to perforation or the need for surgery. On the other hand, in cases where such ultrasound findings are absent, clinicians may be more confident in ruling out evolving NEC, potentially reducing unnecessary antibiotic use, minimizing NPO duration, and avoiding prolonged hospital stays. Thus, ultrasound not only aids in early diagnosis but also supports more judicious, individualized clinical decision-making.

### 5.7. Cost-Effectiveness and Feasibility

Recent studies in LMICs by Chetan et al. and others highlight the growing recognition of abdominal ultrasound (AUS) as a cost-effective diagnostic tool for NEC in neonates [50,51]. Chetan et al. demonstrated that POCUS could reduce imaging delays, enhance bedside decision-making, and limit reliance on radiography, leading to reduced procedural costs. Nagpal et al. emphasized the affordability and practicality of AUS in Indian neonatal intensive care units, noting its potential to lower diagnostic costs in private, out-of-pocket healthcare settings. However, they also pointed to challenges such as limited skilled operators and inconsistent infrastructure. Collectively, these studies support the economic promise of AUS but emphasize that its cost-effectiveness varies significantly based on context. Health system structure, insurance models, and care settings (tertiary vs. community hospitals) influence both feasibility and economic outcomes. Thus, while promising, ultrasound’s widespread adoption as a first-line diagnostic tool for NEC requires more detailed, context-specific economic evaluations, particularly across diverse healthcare infrastructures in both high-income and low- to middle-income countries [50,51].

### 5.8. Limitations

This scoping review was designed to provide a broad overview of the literature on ultrasound in the diagnosis and management of NEC; however, several limitations must be acknowledged. First, the heterogeneity in study designs, populations, ultrasound protocols, and outcome measures across the included studies limited our ability to conduct a formal meta-analysis or quantitatively compare findings. Second, although we employed a comprehensive search strategy across multiple databases, there is a possibility that relevant studies published in non-indexed journals or in languages other than English were inadvertently excluded. Third, the inclusion of both retrospective and prospective studies—many from single centers—introduces potential selection and publication biases. Furthermore, the evolving nature of ultrasound technology and its operator-dependent characteristics, particularly in the context of point-of-care applications, may impact the reproducibility and generalizability of findings. Lastly, the review includes studies with variable reporting of interobserver reliability and long-term clinical outcomes, limiting the strength of conclusions regarding prognostic value. Despite these limitations, this review offers valuable insights into current evidence and identifies key areas for future research and standardization.

### 5.9. Future Directions

Future directions in bowel ultrasound for NEC lie in technological innovation, risk stratification, and standardized implementation. Emerging modalities like UHFUS, operating at 30–100 MHz, offer unparalleled resolution of bowel wall microstructures, enabling earlier detection of ischemic changes and perfusion deficits well before they appear on conventional imaging. The integration of artificial intelligence is set to further revolutionize this space—AI platforms such as RFImageNet and deep learning models that incorporate grayscale imaging, Doppler data, and clinical metadata can enhance diagnostic accuracy, reduce interobserver variability, and support real-time bedside decision-making. Large-scale clinical trials in NICUs will be essential to validate these AI-driven tools and assess their impact on clinical outcomes. Simultaneously, incorporating fetal and early postnatal Doppler studies, especially SMA flow and cerebral Doppler indices, may enable risk stratification before feeding initiation in growth-restricted or hypoxic neonates. Embedding these indices into NEC risk protocols could significantly reduce disease burden. However, the successful adoption of these advancements hinges on robust training, credentialing, and governance frameworks. Institutions must prioritize standardized protocols, clearly defined scopes of practice, competency-based training, and interdisciplinary quality assurance—ensuring that bowel ultrasound becomes a safe, reliable, and transformative tool in neonatal intensive care.

### 5.10. Conclusions

This scoping review and advancement in bowel imaging call for a paradigm shift in neonatal practice—towards early, bedside, non-invasive assessment using bowel ultrasound to detect, predict, and prevent the progression of NEC, thus decreasing morbidity and mortality and improving clinical outcome. Although abdominal radiographs remain the main workhorse modality for NEC diagnosis and confirmation, multiple studies have highlighted the capacity of US to detect features not otherwise seen on radiographs, such as bowel wall integrity, bowel and mesenteric perfusion, peristalsis, and the presence and nature of fluid collections. It also highlights the evolving understanding of NEC, including transfusion-associated NEC and post-surgical gut injury, which represent critical components of the broader disease spectrum. The growing body of neonatal-specific evidence, coupled with advancements in imaging technology, artificial intelligence integration, standardized protocols, and structured training, positions bowel ultrasound as a transformative tool in neonatal care.

## Figures and Tables

**Table 1:** Study designs included in the review and their clinical relevance in the literature.

Study Type	Number of Studies N (%)	Clinical Relevance
RCTs	21 (20.7%)	High-level evidence on ultrasound efficacy
Prospective Cohort	24 (23.7%)	Longitudinal NEC progression data
Retrospective Studies	6 (5.9%)	Assessed ultrasound’s real-world clinical impact
Case-Control	15 (14.8%)	Compared ultrasound findings in NEC vs. non-NEC neonates
Systematic Reviews/Meta-Analyses	10 (9.9%)	Synthesized multi-study data
Observational and Descriptive	25 (25%)	Examined emerging ultrasound techniques

Abbreviations: RCT, randomized controlled trial; NEC, necrotizing enterocolitis.

**Table 2:** Gestational age-specific ultrasound findings in NEC with diagnostic performance metrics.

Gestational Age	Common Ultrasound Findings	Sample Size (n)	Diagnostic Accuracy
<28 weeks (Extremely Preterm)	Thickened bowel wall, absent peristalsis, portal venous gas	45 studies (~2200 infants)	Sensitivity 85–90%; Specificity 80–85%
28–32 weeks (Very Preterm)	Bowel wall thickening, persistent bowel dilatation, variable Doppler	35 studies (~1700 infants)	Sensitivity 78–85%; Specificity 75–80%
>32 weeks (Late Preterm and Term)	Bowel thickening, portal venous gas, free peritoneal fluid	20 studies (~950 infants)	Sensitivity 70–75%; Specificity 70–78%

Ultrasound findings in NEC vary by gestational age, reflecting differences in gut maturity and disease expression. Sample sizes and diagnostic accuracy values are derived from the reviewed literature across 100+ studies.

**Table 3:** Comparative diagnostic value of key ultrasound features in NEC relative to abdominal radiography.

Ultrasound Feature	Clinical Relevance	Comparison with X-Ray
Thickened Bowel Wall (>2.5–3 mm)	Indicates early inflammation	X-ray lacks specificity
Pneumatosis Intestinalis	More sensitive than X-ray in detecting intramural gas	Often missed on early X-ray
Reduced/Absent Peristalsis	Marker of NEC progression	X-ray does not assess motility
Portal Venous Gas	Associated with severe NEC and poor prognosis	Detectable earlier than on X-ray
Free Intraperitoneal Fluid	Suggests advanced disease or perforation	X-ray detects perforation late
Bowel Wall Perfusion (Doppler US)	Identifies ischemia, necrosis	Not available with X-ray

**Table 4:** Summary of key studies on ultrasound in the diagnosis and management of NEC.

Study (Author, Year)	Study Design	Population	Key Finding
Kim et al., 2005 [17]	Prospective cohort	Neonates with early-stage NEC	Ultrasound detected bowel wall thickening earlier than X-ray
Guang et al., 2019 [20]	Prospective study	Preterm neonates	Abnormal SMA Doppler flow associated with NEC developpment
Jacobsen et al., 2024 [32]	Feasibility study	Infants	UHFUS visualized intestinal microvasculature and detected early NEC
Yao et al., 2024 [33]	Comparative diagnostic study	Neonates undergoing ultrasound	AI-assisted ultrasound improved diagnostic accuracy by 15%
Wu et al., 2024 [34]	AI model development	NEC-suspected neonates	Deep learning model outperformed traditional diagnostics
Duci et al., 2022 [18]	Fetal Doppler evaluation	Growth-restricted fetuses	AREDF in SMA predicted later NEC development
Patel et al., 2012 [4]	Systematic review	Very-low-birth-weight infants	POCUS had higher sensitivity than radiography in NEC diagnosis
Muchantef et al., 2013 [24]	Retrospective cohort	Neonates with NEC	Ultrasound detected pneumatosis intestinalis earlier than radiography
Lazow et al., 2021 [21]	Multivariable predictive model	NEC patients undergoing surgery	Ultrasound findings contributed to surgical risk scoring
Zarei et al., 2024 [29]	Prospective observational	Neonates with suspected NEC	Free fluid on ultrasound predicted need for surgery
May et al., 2023 [16]	Expert review	Preterm and term neonates	Identified critical sonographic findings to optimize NEC imaging
Singh et al., 2023 [25]	Translational review	Preterm neonates	Described gestational age-dependent sonographic profiles of NEC

Abbreviations: SMA—Superior Mesenteric Artery; AREDF—Absent or Reversed End-Diastolic Flow; UHFUS—Ultra-High-Frequency Ultrasound; POCUS—Point-of-Care Ultrasound.

## Supplementary Materials

The following supporting information can be downloaded at https://www.mdpi.com/article/10.3390/diagnostics15151852/s1, Table S1: Preferred Reporting Items for Systematic reviews and Meta-Analyses extension for Scoping Reviews (PRISMA-ScR) Checklist. Ref. [52] is cited in the Supplementary Materials file.
