# Supplementary material for: Ultrasound for the Early Detection and Diagnosis of Necrotizing Enterocolitis: A Scoping Review of Emerging Evidence
Source: Diagnostics (Basel). 2025 Jul 23;15(15):1852. doi: 10.3390/diagnostics15151852 (PMC12346292; doi:10.3390/diagnostics15151852)
Supplement: Supplementary file 1 [file diagnostics-15-01852-s001.zip › diagnostics-3631288-supplementary.pdf]

Preferred Reporting Items for Systematic reviews and Meta-Analyses extension for Scoping Reviews (PRISMA-ScR) Checklist

SECTION	ITEM	PRISMA-ScR CHECKLIST ITEM	REPORTED ON PAGE #
**TITLE**			
Title	1	Identify the report as a scoping review.	yes
**ABSTRACT**			
Structured summary	2	Provide a structured summary that includes (as applicable): background, objectives, eligibility criteria, sources of evidence, charting methods, results, and conclusions that relate to the review questions and objectives.	yes
**INTRODUCTION**			
Rationale	3	Describe the rationale for the review in the context of what is already known. Explain why the review questions/objectives lend themselves to a scoping review approach.	yes
Objectives	4	Provide an explicit statement of the questions and objectives being addressed with reference to their key elements (e.g., population or participants, concepts, and context) or other relevant key elements used to conceptualize the review questions and/or objectives.	yes
**METHODS**			
Protocol and registration	5	Indicate whether a review protocol exists; state if and where it can be accessed (e.g., a Web address); and if available, provide registration information, including the registration number.	This scoping review is currently in the process of being registered in a publicly accessible repository. The registration, once complete, will include the review's objectives, eligibility criteria, search strategy, and data charting methodology. While the registration is not yet finalized, the conduct of this review has followed the PRISMA-ScR checklist and methodological frameworks established by Arksey and O'Malley, Levac et al., and the Joanna Briggs Institute. A registration identifier and link will be provided in an updated version of the manuscript upon approval
Eligibility criteria	6	Specify characteristics of the sources of evidence used as eligibility criteria (e.g., years considered, language, and publication status), and provide a rationale.	Studies were included if they examined the role of ultrasound (abdominal, bowel, intestinal, or Doppler) in the diagnosis of necrotizing enterocolitis (NEC) in neonates. Eligible study types included randomized controlled trials (RCTs), observational studies, case series, systematic reviews, and meta-analyses. Only studies published in peer-reviewed journals between the years 2000 and 2025 were considered, in order to capture developments in neonatal ultrasound over the past two decades, including the emergence of point-of-care and Doppler-based modalities. Articles were included if published in English or if an English translation of the abstract was available. Grey

SECTION	ITEM	PRISMA-ScR CHECKLIST ITEM	REPORTED ON PAGE #
			literature, editorials, and case reports involving fewer than five patients were excluded to ensure data consistency and clinical relevance
Information sources*	7	Describe all information sources in the search (e.g., databases with dates of coverage and contact with authors to identify additional sources), as well as the date the most recent search was executed.	A comprehensive literature search was conducted across four major electronic databases: PubMed (MEDLINE), Embase, the Cochrane Library, and Google Scholar. The databases were searched for studies published between January 1, 2000, and February 29, 2024, to capture advancements in ultrasound technology relevant to the diagnosis of necrotizing enterocolitis (NEC) over the past two decades. Additional sources were identified through manual screening of the reference lists of eligible articles and relevant reviews. No restrictions were placed on publication status beyond peer-reviewed availability. The most recent database search was completed on February 29, 2024
Search	8	Present the full electronic search strategy for at least 1 database, including any limits used, such that it could be repeated.	The following search strategy was used in **PubMed (MEDLINE)** to identify relevant studies on the use of ultrasound in the diagnosis of necrotizing enterocolitis (NEC) in neonates. The search was limited to articles published between **January 1, 2000, and February 29, 2024**, and included only articles in English or with English-language abstracts.<br><br>**PubMed Search Strategy:**<br><br>`["Enterocolitis, Necrotizing"[MeSH Terms] OR "necrotizing enterocolitis" OR "NEC")`<br>`AND`<br>`("Infant, Newborn"[MeSH Terms] OR neonate* OR "preterm infant" OR "premature infant")`<br>`AND`<br>`("Ultrasonography"[MeSH Terms] OR "Ultrasonography, Doppler"[MeSH Terms] OR ultrasound OR "Point-of-Care Systems")`<br>`AND`<br>`("Intestines"[MeSH Terms] OR bowel OR intestinal)`<br>`AND`<br>`("Diagnosis"[MeSH Terms] OR "Monitoring, Physiologic"[MeSH Terms] OR assessment OR prognosis)`<br>`Filters: Publication date from 2000/01/01 to 2024/02/29; English]`<br><br>This search was designed to identify all studies that examined ultrasound (including Doppler and point-of-care ultrasound) for NEC diagnosis in neonates and was supplemented by hand-searching reference lists of relevant articles.
Selection of sources of evidence†	9	State the process for selecting sources of evidence (i.e., screening and eligibility) included in the scoping review.	The selection process for sources of evidence followed a three-step approach. First, two independent reviewers conducted **title and abstract screening** of all records retrieved from the electronic databases. Articles deemed potentially relevant were advanced to the second stage for **full-text review**,

SECTION	ITEM	PRISMA-ScR CHECKLIST ITEM	REPORTED ON PAGE #
			during which inclusion and exclusion criteria were applied to assess eligibility. In the third stage, eligible full-text articles underwent **data extraction and charting** using a standardized template. Disagreements at any stage were resolved through discussion and consensus, with a third reviewer available for arbitration if needed. The overall study selection process is summarized in the PRISMA flow diagram (Figure 1)
Data charting process‡	10	Describe the methods of charting data from the included sources of evidence (e.g., calibrated forms or forms that have been tested by the team before their use, and whether data charting was done independently or in duplicate) and any processes for obtaining and confirming data from investigators.	A standardized data extraction template was developed to chart relevant information from the included studies. The charting form was pilot-tested on a subset of five articles to ensure clarity, consistency, and comprehensiveness. Data charting was performed **independently and in duplicate** by two reviewers IB AND RS, to minimize bias and errors. Extracted variables included study design, population characteristics, ultrasound modality and findings, comparison with other imaging techniques, and clinical outcomes. Discrepancies between reviewers were resolved through discussion, and if consensus could not be reached, a third reviewer adjudicated. No contact with original investigators was required for additional data clarification during the charting process.
Data items	11	List and define all variables for which data were sought and any assumptions and simplifications made.	• **Study Identification:** Author(s), year of publication, country of study<br>• **Study Design:** Type of study (e.g., randomized controlled trial, cohort, case-control, case series, systematic review)<br>• **Population Characteristics:** Gestational age, birth weight, number of neonates included, severity of NEC<br>• **Ultrasound Modality:** Type of ultrasound used (e.g., conventional, Doppler, point-of-care, ultra-high-frequency)<br>• **Ultrasound Indication:** Diagnostic, screening, monitoring, or prognostic use<br>• **Key Sonographic Findings:** Bowel wall thickening, pneumatosis intestinalis, absent peristalsis, portal venous gas, free peritoneal fluid, perfusion abnormalities<br>• **Comparison Imaging Modalities:** Radiographs, CT, MRI, or other comparative imaging tools used<br>• **Outcomes Assessed:** NEC stage, need for surgical intervention, diagnostic accuracy, timing of diagnosis, neurodevelopmental outcomes<br>• **Additional Features:** Use of adjuncts like biomarkers (e.g., fecal calprotectin), AI or machine learning tools, follow-up duration<br><br>**Assumptions and Simplifications:**<br><br>• When numerical values (e.g., gestational age ranges or NEC severity scores) were not explicitly reported, categorical groupings (e.g., “extremely preterm”) were used based on standard neonatal definitions.<br>• In studies where ultrasound modality or timing was not clearly differentiated, the

SECTION	ITEM	PRISMA-ScR CHECKLIST ITEM	REPORTED ON PAGE #
			most dominant or primary use reported by the authors was recorded.<br>• If only abstract-level data were available but met inclusion criteria, they were included with annotations regarding data completeness.
Critical appraisal of individual sources of evidence§	12	If done, provide a rationale for conducting a critical appraisal of included sources of evidence; describe the methods used and how this information was used in any data synthesis (if appropriate).	Although critical appraisal is not mandatory in scoping reviews, we conducted a structured appraisal of included studies to assess methodological quality and the strength of evidence. The **Medical Education Research Study Quality Instrument (MERSQI)** was used for this purpose, as it allows evaluation across six domains: study design, sampling, type of data, validity of evaluation instruments, data analysis, and outcomes. This tool was selected due to its applicability to a variety of observational and interventional study designs commonly seen in neonatal research.<br><br>The appraisal was performed independently by two reviewers, and results were used to provide context for interpreting the robustness of findings rather than for inclusion/exclusion decisions. No studies were excluded based on quality scores alone, in line with the scoping review methodology. Instead, critical appraisal findings informed the qualitative synthesis and identification of areas requiring higher-quality evidence
Synthesis of results	13	Describe the methods of handling and summarizing the data that were charted.	The charted data were synthesized using **descriptive and thematic analysis** methods. Studies were first grouped by major characteristics such as study design, population type, and ultrasound modality. Quantitative variables (e.g., frequency of sonographic findings, study distribution across gestational age groups) were tabulated and reported as counts and percentages where applicable.<br><br>Thematic synthesis was then applied to identify recurring **patterns, clinical insights, and emerging concepts** related to the diagnostic role of ultrasound in NEC. These themes included: gestational age-specific sonographic findings, comparison with radiographs, perfusion assessment via Doppler, and innovations such as AI and ultra-high-frequency ultrasound. Summaries were presented in both **narrative form and structured tables/figures** to facilitate clarity and comparison across studies. The findings were not pooled statistically, in accordance with scoping review methodology
**RESULTS**			
Selection of sources of evidence	14	Give numbers of sources of evidence screened, assessed for eligibility, and included in the review, with reasons for exclusions at each stage, ideally using a flow diagram.	Click here to enter text.A total of **327 records** were identified through database and manual searches. After removing **140 duplicates**, **187 unique records** remained for screening. Following title and abstract screening, **53 records** were excluded for not meeting the inclusion criteria, leaving **134 full-text articles** for eligibility assessment. Of these, **33 articles** were excluded due to reasons such as inappropriate study design, population mismatch, or absence of relevant ultrasound-based outcomes.

SECTION	ITEM	PRISMA-ScR CHECKLIST ITEM	REPORTED ON PAGE #
			Ultimately, **101 studies** were included in the final scoping review.<br><br>The full selection process is visually summarized in the **PRISMA-ScR flow diagram (Figure 1)**, including reasons for exclusion at the full-text review stage
Characteristics of sources of evidence	15	For each source of evidence, present characteristics for which data were charted and provide the citations.	Added as word file Appendix 2
Critical appraisal within sources of evidence	16	If done, present data on critical appraisal of included sources of evidence (see item 12).	As noted in Item 12, critical appraisal was conducted using the **Medical Education Research Study Quality Instrument (MERSQI)** across all included empirical studies (excluding systematic reviews). The MERSQI evaluates six domains: study design, sampling, data type, validity of assessment tools, data analysis, and outcomes.<br><br>Among the 91 original studies (excluding 10 reviews), MERSQI scores ranged from **8.5 to 15 out of 18**, with a **mean score of 12.1 (SD = 1.9)**, indicating moderate to high methodological quality across the evidence base. The strongest domains across studies were **study design** and **data analysis**, while the weakest were **instrument validity** and **generalizability** due to small, single-center samples.<br><br>These appraisal findings informed the qualitative interpretation of results and helped contextualize evidence strength by identifying where data were most robust versus where further high-quality studies are needed
Results of individual sources of evidence	17	For each included source of evidence, present the relevant data that were charted that relate to the review questions and objectives.	For each of the 101 included studies, the following data elements were extracted and mapped to the core objectives of the scoping review:<br><br>• **Diagnostic Utility of Ultrasound:** Key sonographic features such as bowel wall thickening, pneumatosis intestinalis, absent peristalsis, portal venous gas, free peritoneal fluid, and altered bowel perfusion patterns were documented. These were examined in the context of their sensitivity, specificity, and clinical relevance for early NEC detection.<br>• **Gestational Age-Specific Insights:** Studies were categorized based on gestational age groups (<28 weeks, 28–32 weeks, >32 weeks), and differences in ultrasound findings and predictive value were analyzed. For instance, extremely preterm infants showed earlier and more severe Doppler abnormalities, while term infants had less specific findings like portal venous gas.<br>• **Comparative Imaging Modalities:** Where available, ultrasound performance was compared with abdominal radiographs and other modalities. Several studies

St. Michael's
Inspired Care.
Inspiring Science.

SECTION	ITEM	PRISMA-ScR CHECKLIST ITEM	REPORTED ON PAGE #
			demonstrated superior sensitivity of ultrasound, particularly for early-stage NEC and dynamic findings like bowel perfusion and peristalsis.<br>• **Clinical Application & Outcomes:** Studies were assessed for whether ultrasound informed clinical decision-making, such as feeding practices, need for surgical consultation, or timing of intervention. A subset of studies also reported long-term outcomes like survival or neurodevelopment in relation to ultrasound-based diagnosis.
Synthesis of results	18	Summarize and/or present the charting results as they relate to the review questions and objectives.	The charted results were synthesized thematically and organized to directly address the core objectives of the scoping review:<br><br>1. **Role of Ultrasound in NEC Diagnosis:** Most studies emphasized the utility of ultrasound for detecting hallmark NEC features such as bowel wall thickening, pneumatosis intestinalis, portal venous gas, and free intraperitoneal fluid. Doppler ultrasound added value by revealing bowel perfusion abnormalities, often preceding clinical deterioration.<br>2. **Comparison with Radiography:** Multiple studies demonstrated that ultrasound had superior sensitivity over abdominal radiographs, particularly in early or atypical presentations. Radiography often failed to detect key findings such as absent peristalsis or early bowel wall changes, reinforcing the complementary role of ultrasound.<br>3. **Gestational Age-Specific Findings:** Ultrasound findings varied with gestational age. Extremely preterm infants showed early ischemic signs on Doppler, while more mature infants often presented with subtle bowel wall changes or isolated portal venous gas.<br>4. **Predictive and Prognostic Utility:** A subset of studies reported that early ultrasound findings, especially perfusion deficits or complex free fluid, were predictive of disease severity and need for surgical intervention. Some also linked early diagnosis via ultrasound with improved long-term neurodevelopmental outcomes.<br>5. **Innovation and Implementation:** Recent studies explored the feasibility of point-of-care ultrasound (POCUS) and the use of AI tools for NEC diagnosis. While promising, these approaches require standardization and further validation.
**DISCUSSION**			
Summary of evidence	19	Summarize the main results (including an overview of concepts, themes, and types of evidence available), link to the review questions and	This scoping review identified and synthesized 101 studies examining the role of ultrasound in the diagnosis and evaluation of necrotizing enterocolitis (NEC) in neonates. The evidence base spans a wide range of study types, including observational studies (cohort, case-control), randomized controlled trials, systematic

SECTION	ITEM	PRISMA-ScR CHECKLIST ITEM	REPORTED ON PAGE #
		objectives, and consider the relevance to key groups.	reviews, and feasibility studies. The literature reflects significant heterogeneity in patient populations, gestational age groups, and ultrasound modalities used.<br><br>Thematic analysis of the included studies revealed five central concepts:<br><br>1. **Ultrasound as a Diagnostic Tool:** Ultrasound was shown to detect hallmark features of NEC—such as bowel wall thickening, pneumatosis, portal venous gas, and free fluid—often earlier than radiographs. The addition of Doppler and real-time motility assessment enhanced its sensitivity.<br>2. **Gestational Age Variability:** Findings consistently indicated that ultrasound features of NEC differ by gestational age. Extremely preterm infants had earlier and more severe Doppler changes, while late preterm and term neonates presented with more subtle or delayed sonographic signs.<br>3. **Clinical Relevance and Outcomes:** Ultrasound-informed diagnosis enabled early intervention in several studies, with some demonstrating improved survival or neurodevelopmental outcomes when NEC was diagnosed sonographically. Sonographic predictors such as absent peristalsis and complex ascites were linked to need for surgery.<br>4. **Comparison to Traditional Imaging:** Across many studies, ultrasound outperformed abdominal radiographs in sensitivity and dynamic information, particularly in early NEC or atypical cases. Radiographs continued to serve as a confirmatory and complementary tool.<br>5. **Innovation and Equity in Access:** Recent advancements include the emergence of point-of-care ultrasound (POCUS), use of AI-based diagnostic tools, and ultra-high-frequency probes. These innovations show promise but require validation, training, and equitable access—especially in resource-limited settings.<br><br>The review's findings are highly relevant to neonatologists, radiologists, sonographers, and NICU leadership teams seeking to improve NEC detection and reduce diagnostic delays. Standardized ultrasound protocols, training programs, and future prospective trials are needed to scale and validate its implementation.
Limitations	20	Discuss the limitations of the scoping review process.	This scoping review has several limitations inherent to the methodology and scope of included studies. First, while the review followed PRISMA-ScR guidelines and included a structured search across multiple databases, there remains a risk of **publication bias** and **language bias**, as only English-language studies and peer-reviewed literature were included. Relevant findings in

SECTION	ITEM	PRISMA-ScR CHECKLIST ITEM	REPORTED ON PAGE #
			non-English publications or grey literature may have been missed.<br><br>Second, **critical appraisal was conducted to assess study quality** using the MERSQI tool, but these assessments did not influence inclusion, in accordance with scoping review methodology. As a result, findings from studies of lower methodological rigor were synthesized alongside higher-quality studies, which may introduce variability in the strength of evidence.<br><br>Third, while data extraction and screening were conducted independently by two reviewers, **no formal review protocol was registered prior to study initiation**, which may limit reproducibility.<br><br>Fourth, due to the **heterogeneity in study designs, populations, and outcome measures**, data synthesis was limited to descriptive and thematic analysis without quantitative pooling or meta-analysis.<br><br>Finally, as a rapidly evolving field, **recent technological innovations in ultrasound** (e.g., ultra-high-frequency ultrasound and AI-assisted interpretation) are still emerging and underrepresented in current literature. This may limit the generalizability of findings across all clinical settings and NICU infrastructures, especially in low-resource environments.
Conclusions	21	Provide a general interpretation of the results with respect to the review questions and objectives, as well as potential implications and/or next steps.	This scoping review demonstrates that ultrasound, particularly when supplemented with Doppler and point-of-care techniques, is an increasingly valuable tool for the early detection and diagnosis of necrotizing enterocolitis (NEC) in neonates. Across 101 included studies, evidence consistently supports ultrasound's ability to visualize pathologic features of NEC—such as bowel wall changes, vascular perfusion, and intraperitoneal fluid—before they become evident on radiographs or clinical examination.<br><br>The findings affirm the original review objectives: (1) ultrasound has distinct diagnostic and prognostic utility in NEC; (2) its performance is superior to traditional imaging modalities in several clinical contexts; and (3) further standardization and training are critical for widespread implementation. Gestational age-specific findings emphasize the importance of tailoring sonographic interpretation to neonatal maturity, and early Doppler changes hold promise as biomarkers for risk stratification.<br><br>From a clinical perspective, the integration of bowel ultrasound into NICU workflows may allow for more timely interventions, reduced surgical NEC, and potentially improved neurodevelopmental outcomes. However, its successful implementation depends on structured training, quality assurance, and interdisciplinary collaboration among neonatologists, radiologists, and sonographers.<br><br>Future research should focus on:

SECTION	ITEM	PRISMA-ScR CHECKLIST ITEM	REPORTED ON PAGE #
			• Large-scale prospective studies to validate sonographic predictors of NEC severity and surgical need;<br>• Development of standardized protocols and image scoring systems;<br>• Evaluation of cost-effectiveness and feasibility in low-resource settings;<br>• Clinical integration of AI tools and ultra-high-frequency ultrasound in routine neonatal imaging.<br><br>In conclusion, bowel ultrasound is poised to play a central role in a precision-guided, non-invasive diagnostic strategy for NEC, with the potential to reduce diagnostic uncertainty and improve care for one of the most devastating conditions in neonatal medicine.
**FUNDING**			
Funding	22	Describe sources of funding for the included sources of evidence, as well as sources of funding for the scoping review. Describe the role of the funders of the scoping review.	This scoping review did not receive any external funding. All activities related to study design, literature search, data extraction, analysis, and manuscript preparation were conducted independently by the authors. The authors declare that no funding body had any role in the design, conduct, interpretation, or reporting of this review.

JBI = Joanna Briggs Institute; PRISMA-ScR = Preferred Reporting Items for Systematic reviews and Meta-Analyses extension for Scoping Reviews.
* Where *sources of evidence* (see second footnote) are compiled from, such as bibliographic databases, social media platforms, and Web sites.
† A more inclusive/heterogeneous term used to account for the different types of evidence or data sources (e.g., quantitative and/or qualitative research, expert opinion, and policy documents) that may be eligible in a scoping review as opposed to only studies. This is not to be confused with *information sources* (see first footnote).
‡ The frameworks by Arksey and O'Malley (6) and Levac and colleagues (7) and the JBI guidance (4, 5) refer to the process of data extraction in a scoping review as data charting.
§ The process of systematically examining research evidence to assess its validity, results, and relevance before using it to inform a decision. This term is used for items 12 and 19 instead of "risk of bias" (which is more applicable to systematic reviews of interventions) to include and acknowledge the various sources of evidence that may be used in a scoping review (e.g., quantitative and/or qualitative research, expert opinion, and policy document).

From: Tricco AC, Lillie E, Zarin W, O'Brien KK, Colquhoun H, Levac D, et al. PRISMA Extension for Scoping Reviews (PRISMAScR): Checklist and Explanation. Ann Intern Med. 2018;169:467–473. doi: 10.7326/M18-0850.
